# Perchloroethylene and Dry Cleaning: It's Time to Move the Industry to Safer Alternatives

**DOI:** 10.3389/fpubh.2021.638082

**Published:** 2021-03-05

**Authors:** Diana M. Ceballos, Katie M. Fellows, Ashley E. Evans, Patricia A. Janulewicz, Eun Gyung Lee, Stephen G. Whittaker

**Affiliations:** ^1^Department of Environmental Health, Boston University School of Public Health, Boston, MA, United States; ^2^Hazardous Waste Management Program in King County, Seattle, WA, United States; ^3^National Institute for Occupational Safety and Health, Respiratory Health Division, Field Studies Branch, Morgantown, WV, United States

**Keywords:** dry-cleaning, chlorinated solvents, human health, safer alternatives, PERC, professional wet cleaning

## Abstract

Perchloroethylene (PERC) is the most common solvent used for dry cleaning in the United States. PERC is a reproductive toxicant, neurotoxicant, potential human carcinogen, and a persistent environmental pollutant. The Environmental Protection Agency is evaluating PERC under the Frank R. Lautenberg Chemical Safety for the 21st Century Act, which amended the Toxic Substances Control Act (amended TSCA), and has mandated that PERC dry cleaning machines be removed from residential buildings. Some local and state programs are also requiring or facilitating transitions to alternative cleaning technologies. However, the potential for these alternatives to harm human health and the environment is not well-understood. This review describes the issues surrounding the use of PERC and alternative solvents for dry cleaning while highlighting the lessons learned from a local government program that transitioned PERC dry cleaners to the safest current alternative: professional wet cleaning. Implications for future public health research and policy are discussed: (1) we must move away from PERC, (2) any transition must account for the economic instability and cultural aspects of the people who work in the industry, (3) legacy contamination must be addressed even after safer alternatives are adopted, and (4) evaluations of PERC alternatives are needed to determine their implications for the long-term health and sustainability of the people who work in the industry.

## Introduction

### Dry Cleaning and the Use of Perchloroethylene

Dry cleaning uses non-aqueous solvents to clean fabrics (1). The first dry cleaning operations in the United States (US) date back to the 1800s when people washed fabrics in open tubs with solvents such as gasoline, kerosene, benzene, turpentine, and petroleum and then hung to dry. In the 1900s, the US started using specialized machines for the dry cleaning process. However, the use of highly flammable petroleum solvents caused many fires and explosions, highlighting the need to find a safer alternative. The dry cleaning industry first introduced Stoddard solvent (less flammable than gasoline) followed by several nonflammable halogenated solvents, such as carbon tetrachloride, trichloroethylene (TCE), trichlorotrifluoroethane, and perchloroethylene (PERC). Beginning in the 1940s, PERC—also known as tetrachloroethylene or PCE—became the most frequently used dry cleaning solvent (1, 2) and continues to be the primary solvent used to dry clean fabrics both in the US (3) and the European Union (EU) (4).

To comply with environmental regulations, dry cleaning machines have evolved through several “generations” to minimize PERC release. The 1st generation machines were “transfer machines,” where cleaned fabrics were manually transferred from the washer to a dryer. Since then, various pollution prevention controls have been implemented through the subsequent generations, culminating in the latest 5th generation machines, which are closed-loop and equipped with refrigerated condensers, carbon absorbers, inductive fans, and sensor-actuated lockout devices (1, 4–6). As the newer generations of machines were introduced, the amount of PERC used was reduced from 300 to 500 g-PERC/kilogram of fabrics (1st generation) to <10 g-PERC/kilogram cleaned garment (5th generation) (4). In many EU countries, dry cleaning machines older than 15 years are typically prohibited—only 5th generation machines are allowed. However, 4th generation machines may be used if best practices (e.g., good housekeeping, optimal machine operation, and recycling) are implemented and they meet EU emission requirements (4, 7). The US EPA's National Emission Standards for Hazardous Air Pollutants (NESHAPS) regulations stipulate that 2nd generation machines must be upgraded to 4th generation, and 3rd generation machines must be retrofitted or upgraded to 4th generation machines; only 4th generation and later machines can be sold, leased, or installed (8).

As of 2017 in the US, there are ~20,600 dry cleaning shops and the industry employs nearly 160,000 workers, with ~80% identifying as a racial or ethnic minority (9, 10). The majority of owners are of Korean ancestry (11). Nationwide, 60–65% of dry cleaners use PERC as their primary solvent (1) and most of the remainder use a high-flashpoint hydrocarbon. Other solvents currently used in the US include butylal, siloxane, liquid carbon dioxide, glycol ethers, and water (professional wet cleaning). In Europe, 60–90% of dry cleaning shops use PERC, depending on the country (4).

### Health and Environmental Impacts of Perchloroethylene

PERC is a respiratory and skin irritant, neurotoxicant, liver and kidney toxicant, and reproductive and developmental toxicant (12–17). PERC is also considered a “potential occupational carcinogen” (18), “likely to be carcinogenic to humans by all routes of exposure” (14, 19), and “probably carcinogenic to humans” (20). Neurotoxicity is the most sensitive non-cancer adverse health effect associated with PERC, with negative outcomes occurring even at low-dose exposures (16). Specifically, chronic (i.e., years) or sub-chronic (i.e., months) PERC exposure in humans has been associated with deficits in color vision and neuropsychological function in both occupational and community exposure studies (16).

A comprehensive review of 109 occupational studies with personal exposure measures estimated a mean exposure to PERC of 59 parts per million (ppm) among dry cleaning workers (2), with <10 ppm for spotters, pressers, and counter clerks and >100 ppm for machine operators. Another study in 2014 in The Netherlands surveyed ambient PERC concentrations for 193 dry cleaning shops before and after implementing a certification program that customers can use to select shops that are more safe and environmental friendly (4). Before the program, about 77% of shops reported 15-min time-weighted average (TWA) airborne concentrations ≥35 ppm. After the program, all shops showed a 15-min TWA of <35 ppm. These reductions were encouraging and below the European Union 15-min TWA limit of 40 ppm. However, decrements in visual reproduction, pattern memory, and pattern recognition were found among 65 workers when exposed to an average TWA concentration of <50 ppm for at least 3 years (21). Decrements on cognitive tests of attention and visual perception were seen in 100 workers with average full-shift TWA exposures of 12 ppm (22). Decrements were also found with cognitive tests of attention, specifically impaired reaction time, and vigilance among 60 workers typically exposed to TWA of 15 ppm (23). Reduced performance on vocal reaction time to visual stimuli was seen among 35 workers with TWA as low as 8 ppm (24). Residents who lived near a dry cleaning shop for an average of 10.6 years (mean indoor air concentration of 0.7 ppm) were found to have reduced cognitive performance on a test of reaction time, vigilance, and visual memory (25).

Numerous communities have been impacted through exposure to PERC. A cluster of communities on Cape Cod, Massachusetts has been extensively studied following years of PERC exposure. In this region, some water pipes were replaced with vinyl-lined asbestos-cement pipes (26). The vinyl lining was applied with a slurry of vinyl resin and PERC. Although it was believed that the PERC would evaporate before installation, subsequent water quality testing revealed that the people living in these communities were being exposed to PERC in their drinking water, ranging from 1.5 to 7,750 μg/L (26, 27). Residents experienced adverse reproductive health outcomes, including delayed time-to-pregnancy (27, 28), increased risk of placental abruptions (27, 28), and an increased risk of congenital malformations (29). Exposure during the prenatal and early childhood period also yielded adverse impacts in adulthood, including reduced performance on neuropsychological tests (26), increased risk of bipolar disorder (27, 30), Post Traumatic Stress Disorder (27, 30), illicit drug use (31, 32), vision problems (33), and certain types of cancer (32, 34, 35). However, no literature was found that describes the regional impact of community PERC exposures through other routes, such as inhalation.

PERC is a persistent pollutant that can contaminate air, soil, groundwater, drinking water, and is potentially toxic to wildlife (13, 30, 31, 34). The recent draft US EPA risk evaluation on PERC found environmental risks to aquatic organisms (36). PERC poses a hazard to environmental aquatic receptors, including aquatic invertebrates, fish, and aquatic plants. The most sensitive species for acute toxicity were two daphnid species, *Ceriodaphnia dubia* and *Daphnia magna*; the acute toxicity value was as low as 2.5 milligrams per liter (mg/L). PERC presents an acute hazard to fish based on the mortality of rainbow trout (the most sensitive species) with acute toxicity values as low as 3.6 mg/L for mortality (i.e., the LC_50_—the concentration required to kill 50% of the population) (37). For chronic exposures, PERC is a hazard to aquatic invertebrates, with a chronic toxicity value of 0.5 mg/L, and a chronic toxicity value of 0.8 mg/L for fish (38). PERC is also a hazard for green microalgae with toxicity values as low as 0.02 mg/L (38).

### Regulations for Perchloroethylene

Since 1988, US workplaces have been regulated by the US Occupational Safety and Health Administration (OSHA) with enforceable occupational exposure limits for PERC of 100 ppm for a full-shift (8-h TWA) and 200 ppm for a ceiling limit (39). The European Union set lower limits than OSHA, with 20 ppm (138 mg/m^3^) for the 8-h TWA and 40 ppm (275 mg/m^3^) for the 15-min short-term TWA (38). PERC emissions have also been regulated since the 1990s under NESHAPS (5). By 2003, the California Air Resources Board (CARB) established its Non-Toxic Dry Cleaning Incentive Program (AB998) to help dry cleaners transition away from PERC (40). In 2007, CARB initiated a phase-out of the use of PERC dry cleaning machines in the State of California by January 1, 2023 (41). This regulatory action by CARB promoted the adoption of new technologies nationwide.

Under the Clean Air Act (in the Final Amendments to Air Toxics Standards for Perchloroethylene Dry Cleaners), the US EPA stipulates that all PERC machines be removed from residential buildings by December 21, 2020, and replaced with non-PERC technology (42). PERC is also one of the first ten chemicals being evaluated by EPA under the Frank R. Lautenberg Chemical Safety for the 21st Century Act (Lautenberg Chemical Safety Act), which amended TSCA (amended TSCA) (43). In 2020, a draft risk evaluation released by the US EPA preliminarily found unreasonable risk to workers, occupational non-users, consumers, bystanders, and the environment from certain uses of PERC, including its use in dry cleaning (36). Consequently, the US EPA may issue a national ban on the use of PERC in dry cleaning by 2021. None of the EU countries have banned the use of PERC in dry cleaning because they considered that the health and safety of dry cleaners is assured by implementing control measures. Although over 90% of dry cleaning shops still use PERC in France, PERC dry cleaning machines will be phased-out in residential areas by 2022 (44).

The Toxics Use Reduction Institute (TURI) ranked the available alternatives against PERC, based on technical, economic, environmental, regulatory, and human health criteria. The alternatives were then placed into one of five groupings, with group 1 being the most preferred and group 5 the least preferred. Professional wet cleaning (i.e., water) was the only group 1 alternative, followed by liquid carbon dioxide (group 2); high flashpoint hydrocarbons, butylal (acetal), and propylene glycol ethers (group 3); siloxane (group 4), and finally *n*-propyl bromide (*n*-PB) (group 5) (45). A comprehensive review of fabric cleaning technologies was also published by investigators at RMIT University, Australia (46) that focused on ecological attributes and sustainability of safe apparel cleaning method alternatives to PERC. This review emphasized professional wet cleaning as the most desirable alternative.

Given the ample evidence of the health and environmental impacts of PERC, as well as the many regulations and policy initiatives that make the case to minimize or eliminate PERC in the US and abroad, the main objectives of this paper are to (1) provide an overview of the state of the knowledge regarding safer alternatives to PERC in dry cleaning, with emphasis on studies related to human exposure and health; (2) highlight efforts to transition away from PERC in dry cleaning in the US and in particular in King County, WA, USA; and (3) discuss the implications for future public health research and policy for PERC in dry cleaning and safer alternatives.

## Discussion

### Alternatives to Perchloroethylene

#### n-Propyl Bromide

The promotion of *n*-PB (also known as 1-bromopropane or 1-BP) is a case study in “regrettable substitution,” which is defined as “the substitution of hazardous substances with substances with similar chemical structure and similar hazard properties or with substances with other effects of similar concern” (38). *n*-PB is the only drop-in substitute for PERC (i.e., it can be used in an existing PERC machine with minor modifications). The other alternatives require investment in expensive new dry cleaning equipment. *n*-PB was marketed as a safer alternative because it does not deplete stratospheric ozone. However, this brominated hydrocarbon is extremely toxic to humans via inhalation and is a potent irritant and neurotoxicant. *n*-PB is also reasonably anticipated to be a human carcinogen (47). In 2008, a case study was published that a dry cleaner located in New Jersey developed neurological symptoms after switching from PERC to *n*-PB (48). Also in New Jersey, exceedances of the ACGIH Threshold Limit Value (TLV) of 10 ppm for *n*-PB (49) were documented in dry cleaning shops that had switched from PERC (50). The authors surmised that leakage of *n*-PB from these machines likely reflected the relatively poor condition of the aging PERC dry cleaning equipment and failure to make needed modifications. Along with PERC, *n*-PB is one of the first ten chemicals being evaluated under the amended TSCA (51).

#### Volatile Methyl Siloxanes

Decamethylcyclopentasiloxane or D5, a volatile methyl siloxane, is a colorless, odorless liquid and is not considered a Volatile Organic Compound (VOC) per state and federal air quality regulations. However, there are concerns about the global environmental distribution of this chemical class (52). Although the Canadian government recognized the environmental persistence of siloxanes, in 2013 it concluded that they do not pose a threat to the environment (53). A chronic toxicity study in female rats suggested that siloxanes caused uterine cancer at the highest concentration (54, 55). However, the study authors concluded that the findings of uterine tumors in rats are not relevant to humans. The 2014 Safety Data Sheet (SDS) reviewed by the New York State Department of Environmental Conservation (NYSDEC) states that “This product is not considered to be a carcinogen by IARC, ACGIH, NTP, or OSHA” (56). In conclusion, although the carcinogenicity data for siloxanes are equivocal, a meta-analysis of the toxicological data presented in the Toxnot hazard screening tool revealed that this chemical class poses a very high hazard for environmental persistence (57).

#### Glycol Ethers

Several glycol ether formulations are available, including dipropylene glycol tert-butyl ethers (DPTB), dipropylene glycol n-butyl ether (DPNB), and propylene glycol t-butyl ether (PGtBE). These are organic and biodegradable solvents with low volatility and a high flashpoint. Brand names include Rynex® and Solvair®. There is limited information about the toxicity of DPNB and DPTB. The California Office of Environmental Health Hazard Assessment (OEHHA) lists PGtBE as a potential carcinogen for consideration under Proposition 65 (58).

#### Butylal

Butylal is marketed by Kreussler GmbH as Solvon K4^TM^ and is part of a relatively new dry cleaning process called System K4^TM^ (59). Solvon K4^TM^ is composed primarily of butylal, which is a diether acetal. Synonyms for butylal include dibutoxymethane, 1-(butoxymethoxy)butane, and formaldehyde dibutyl acetal. According to the manufacturer, n-butyl alcohol (1-butanol) and formaldehyde are present at <0.5 and <0.05%, respectively (60). While butylal is reportedly stable at pHs between 4 and 14, the solvent might theoretically hydrolyze in the dry-cleaning machine to create formaldehyde in the presence of acid and heat.

Although the solvent is reportedly slightly biodegradable, there is little published information concerning its aquatic toxicity (61). An LC_50_ for Solvon K4^TM^ of 45.7 mg/L was derived in a 96-h static renewal fish bioassay with juvenile rainbow trout (62); PERC was found to be greater than ten times more toxic than Solvon K4^TM^ in the same bioassay (PERC LC_50_ = 3.61 mg/L) (37).

The available data on butylal's effects on human health are limited to dermal and oral exposures (63). TURI concluded that toxicological data are lacking for this solvent, rendering the human health assessment incomplete (45).

Inhalation exposure assessment of dry cleaners using Solvon-K4^TM^ revealed that the highest exposures (up to 1.9 ppm of butylal) were during pressing, spot cleaning, as well as loading and unloading the dry cleaning machine (64, 65). The operator wore leather gloves to clean out the still bottoms and butylal was detected in all four dermal samples from the operator's gloved hands. Although no occupational exposure limits exist for butylal, there is a risk of skin irritation (66). When control banding techniques were used to assess inhalation and dermal risks (64), the exposures noted at these shops suggested that better controls were needed. Further, inhalation of formaldehyde and butanol (potential hydrolysis products of butylal) were also assessed but exposures were either very low or not detected.

#### High-Flashpoint Hydrocarbons

These petroleum-based solvents are composed of aliphatic hydrocarbons and have relatively high flammability (flashpoints of 140–150°F) and volatility. Examples of these solvents include Exxon-Mobil's DF-2000^TM^ and Chevron Phillips' EcoSolv®. They are classified as synthetic hydrocarbons and are produced using specific feedstocks and process conditions that yield isoparaffins that are low in impurities (67). Chemical analysis of DF-2000^TM^ and EcoSolv® confirmed that they contain between 11 and 15 carbons as their primary structural backbone (i.e., C11 to C15) and do not contain detectable levels of benzene or other hazardous aromatic hydrocarbons (64, 65, 68, 69). Both products are essentially insoluble in water and failed to elicit mortality to juvenile rainbow trout at the highest test concentrations (5,000 mg/L for DF-2000^TM^ and 100 mg/L for EcoSolv®). In the DF-2000^TM^ bioassay, the measured concentration in the test vessel containing 5,000 mg/L of solvent was less than the reporting detection limit of 235 micrograms per liter (μg/L) (68, 69).

The Hazardous Waste Management Program in King County, WA, USA (Haz Waste Program) reviewed these products and concluded that the uncertainty concerning the toxicological properties of this chemical class reflects: (1) the inclusion of diverse products by some investigators in the category of “hydrocarbon dry cleaning solvents,” some of which may contain benzene and other hazardous substances (e.g., Stoddard solvent), and (2) the inadequacy of Chemical Abstract Service (CAS) numbers to uniquely identify specific products within this chemical class; the assigned CAS numbers apply primarily to feedstocks, rather than the finished products (68). However, the authors concluded that there are data gaps in their toxicity and bioaccumulative potential. Because high-flashpoint hydrocarbons are regarded as VOCs by state and federal agencies, they can have adverse impacts on ambient air quality (68).

Inhalation and dermal exposure assessment of dry cleaners using DF-2000^TM^ revealed that the highest personal airborne exposures occurred when workers loaded and unloaded the dry cleaning machines and pressed dry cleaned fabrics. The highest detected full-shift air concentration was 21 milligrams/cubic meter (mg/m^3^), which is considerably lower than the occupational exposure limit of 300 mg/m^3^ (i.e., the GESTIS International 8-h Limit Value for CAS number 64742-48-9) (70). The greatest opportunity for dermal exposure occurred when solid waste (still bottoms) was removed from the dry cleaning machine for disposal; DF-2000^TM^ was detected at very low levels in two of the six dermal samples from the dry cleaners' gloved hands (64, 65).

#### Liquid Carbon Dioxide

This technology combines carbon dioxide with specialized detergents under high pressure (700 psi) and is considered to be non-toxic, non-flammable, non-corrosive, and environmentally safe (46). However, the high cost of the initial capital investment in addition to the ongoing costs for specialized detergents and maintenance has made this technology prohibitively expensive for most dry cleaners (45).

#### Professional Wet Cleaning (PWC)

In PWC, fabrics are cleaned with water and detergent in a computer-controlled washing machine with multiple fabric-specific cleaning programs. In advanced PWC machines, additional products are added to the washing drum, depending on the type of fabric being cleaned. These products protect fibers during drying, prevent dye bleeding, provide suppleness to leather, etc. The washed fabrics are then placed in a specialized dryer equipped with moisture sensors to ensure that fabrics do not shrink after excessive drying. In contrast to PERC and most other solvent-based dry cleaning methods, PWC does not generate hazardous organic solvent waste (71). Another benefit of PWC is that the ancillary process chemicals, including detergents and spot cleaners, are typically less hazardous than those used in PERC and high-flashpoint hydrocarbon dry cleaning (72–74).

Although PWC has been used as an alternative to PERC in the US for over two decades, the dry cleaning community has been slow to adopt this technology. The benefits of PWC and the industry pressures and other factors that have prevented wider adoption of this technology were described as early as 2001 (75). Others have also documented the considerable health, environmental, and economic benefits of using PWC relative to PERC (4, 45, 46, 76–78).

### Promoting Safer Alternatives to PERC in the United States

Several jurisdictions have encouraged or mandated a transition away from PERC. The State of California provided $10,000 grants to PERC dry cleaners to transition to non-toxic and non-smog forming technologies such as PWC and liquid carbon dioxide (40). The Commonwealth of Massachusetts offered grants of up to $10,000 to transition away from PERC (79). The City of Minneapolis banned the use of PERC and became the first PERC-free city in the nation in January 2018 (80). The City of Philadelphia extended the US EPA phase-out of PERC dry cleaning operations located in residential buildings to include hospitals, daycares, schools, health clinics, community centers, and recreation areas (81). The City of New York, among other actions, required all dry cleaners to post the type of chemicals they use via public “right to know” legislation (82).

#### Promoting Safer Alternatives to PERC in King County, WA, USA

##### Learning From the Industry

The Haz Waste Program has provided technical and financial assistance to the local dry cleaning community since the 1990s. These efforts have included technical and financial assistance with pollution prevention, enrollment in an environmental recognition program (“EnviroStars”), grants for alternative dry cleaning equipment, exposure monitoring, and sponsorship of local dry cleaning association meetings (71). The program has also conducted many interviews and focus groups with dry cleaning business owners and vendors.

In 2010, a countywide survey (6, 83) revealed that 69% of dry cleaners in King County, WA, USA, were still using PERC and that cost was the principal barrier to shops adopting safer technologies. Other key findings included that 80% of shops were owned and operated by immigrants from South Korea. Subsequent field visits revealed that when shops had employees, they were typically people of Latin American descent (84). From an equity and social justice perspective, the program considered this to be a vulnerable and underserved population that requires particular protection from the adverse health effects associated with PERC and other hazardous substances. The median age of PERC dry cleaning machines in King County, WA, USA was 18 years, which exceeds their expected service life of ~15 years (6, 83). Older machines can be prone to leaks and other mechanical problems.

##### Selection of the Preferred Alternatives

The Haz Waste Program reviewed the available alternatives to select a technology to promote in King County, WA, USA. Part of this review process included evaluating safer alternatives that had been adopted elsewhere in the US. For example, the NYSDEC has reviewed most of the common alternative dry cleaning solvents, and all but *n*-PB are currently approved for use in New York State (85).

Upon discussing alternatives to PERC with the King County dry cleaning community, their preferred system was high-flashpoint hydrocarbon (71). In King County, WA, USA the primary alternative technologies available to local dry cleaners are high-flashpoint hydrocarbon and butylal (see Table 1). The Haz Waste Program was not aware of any local shops that were using *n*-PB, siloxanes, glycol ethers, or liquid carbon dioxide. By 2010, 27% of shops had already adopted high-flashpoint hydrocarbon in King County, WA, USA (6, 83). Stated reasons for doing so included the belief that high-flashpoint hydrocarbon can clean all fabrics and is similar enough to PERC that little training is required for owners and staff, resulting in less downtime.

**Table 1:** The most prevalent cleaning systems in King County, WA, USA.

**Cleaning system**	**Solvent type**	**Example products (manufacturer)**	**Flashpoint**^a^****	**LC_**50**_ (fish bioassay)**^b^****	**Generates hazardous waste?**^c^****
PERC	Chlorinated hydrocarbon	PerSec® (R.R. Streets & Co., Inc.)	None	3.61 mg/L	Yes
Butylal	Diether acetal	Solvon K4^TM^ (Kreussler GmbH)	143.6°F	45.7 mg/L	Yes
High-flashpoint hydrocarbon	Isoparaffin (C11-C15)	DF-2000^TM^ (Exxon-Mobil)	142°F	>5,000 mg/L	Yes
		EcoSolv® (Chevron Phillips)	144°F	>100 mg/L	Yes
PWC	Water	Not applicable	Not applicable	Not applicable	No

a *From applicable Safety Data Sheets (63, 86–88)*.

b *96-h juvenile rainbow trout bioassays (37, 69, 72, 89)*.

c *Organic solvent waste, per King County Ecology's Dangerous Waste Regulations for Washington State (72, 73, 89–91)*.

Before the advent of the latest PWC technology, the Haz Waste Program provided financial incentives for shops to transition principally to high-flashpoint hydrocarbon (see Table 2). However, dry cleaners continued to use hazardous spot cleaning products from their PERC operations and the solid waste stream (i.e., still bottoms) was determined to be a dangerous waste, based on its toxicity in a fish bioassay (72, 73, 89, 90). The Haz Waste Program also witnessed contamination of high-flashpoint hydrocarbon machines and waste streams from the use of process chemicals that contain chlorinated hydrocarbons (i.e., PERC and TCE).

**Table 2:** Summary descriptors of dry cleaning shop transitions to safer dry cleaning alternatives in King Country, WA, USA.

**Alternative dry cleaning system**	**Disadvantages of alternative system**	**Advantages of alternative system**	**Dates transitioned by King County**	**Financial incentive from King County**	**Number of shops transitioned with King County assistance**
Solvon K4	• Generates hazardous waste • Potential formaldehyde byproduct • Solvent used occasionally for spot cleaning fabrics • Strong odor • More flammable than PERC (Class IIIA solvent) • Toxicity poorly characterized • Proprietary solvent from a single manufacture • Relatively expensive	• Less toxic than PERC • Safer spotting chemicals • Similar to PERC cleaning process	None	None	None
High-flashpoint hydrocarbon	• Generates hazardous waste • More flammable than PERC (Class IIIA solvent) • Often used with legacy spotting chemicals • Machines inadvertently contaminated with PERC process chemicals	• No odors • Relatively inexpensive solvent • Available from multiple manufacturers • Similar to PERC cleaning process • Toxicity relatively well-characterized	2012–2014	$10,000–15,000	9
PWC	• Skepticism of dry cleaners • Relatively expensive equipment • Modern tensioning equipment needed • New cleaning system—training needed • Customer concerns • Adequate boiler needed	• No hazardous waste • No odors • Reduced resource usage • Lower utility bills • Provided spot cleaners are low toxicity	2012–2014 2018–2020	$10,000–15,000 $20,000	2 27

The butylal process was also considered for promotion by the Haz Waste Program. An added benefit of this system is that it includes a suite of spot cleaning chemicals that appear to be relatively safe (72). However, butylal was also ultimately rejected because of uncertainties concerning the solvent's toxicological properties and that the still bottoms were determined to be extremely hazardous waste (72, 89, 90).

The program learned that the local dry cleaning community was skeptical about the ability of PWC to clean all “dry clean only” fabrics, especially wools and silks. Concerns were also expressed about potential shrinkage and the manual labor required to measure garments before cleaning to stretch them back to their original dimensions. However, a new generation of PWC equipment appeared in approximately 2017, and program staff witnessed the successful cleaning of wool dress suits and silk garments in three shops. Interviews with these shop owners and their equipment vendors led the program to conclude that PWC had become a viable alternative to PERC dry cleaning and that it would promote the adoption of PWC exclusively. The program concluded that the alternative organic solvents should no longer be considered for the financial incentive initiative because: (1) they are more flammable than PERC, (2) they generate hazardous waste, and (3) there are numerous uncertainties and data gaps associated with the toxicology of some of these products. The selection of PWC as the preferred alternative represented a precautionary approach to help avoid a regrettable substitution.

##### Implementing a Safer Alternatives Strategy

Having selected PWC as the preferred alternative to PERC, the Haz Waste Program reviewed the approaches used by other jurisdictions to promote adoption. These included financial assistance, equipment demonstrations, bans, and signage requirements (71). These approaches were evaluated against four criteria: (1) human health and environmental impact, (2) financial impact on dry cleaner owners and workers, (3) feasibility, and (4) implementation cost. The program selected the strategy of financial incentives because it had a high likelihood of improved human health/environment protection, minimized the financial impact to dry cleaners, and limited the risk of “regrettable substitutions.” The program chose not to implement a ban because the US EPA was reviewing PERC under the amended TSCA and its decision would likely preempt any regulations introduced in King County, WA, USA. Generally, state and local action on a chemical is preempted when EPA has acted by either finding a chemical to be safe or by regulating a chemical to address identified risks. State action is also temporarily “paused” when EPA is evaluating a chemical.

**Figure 1:**
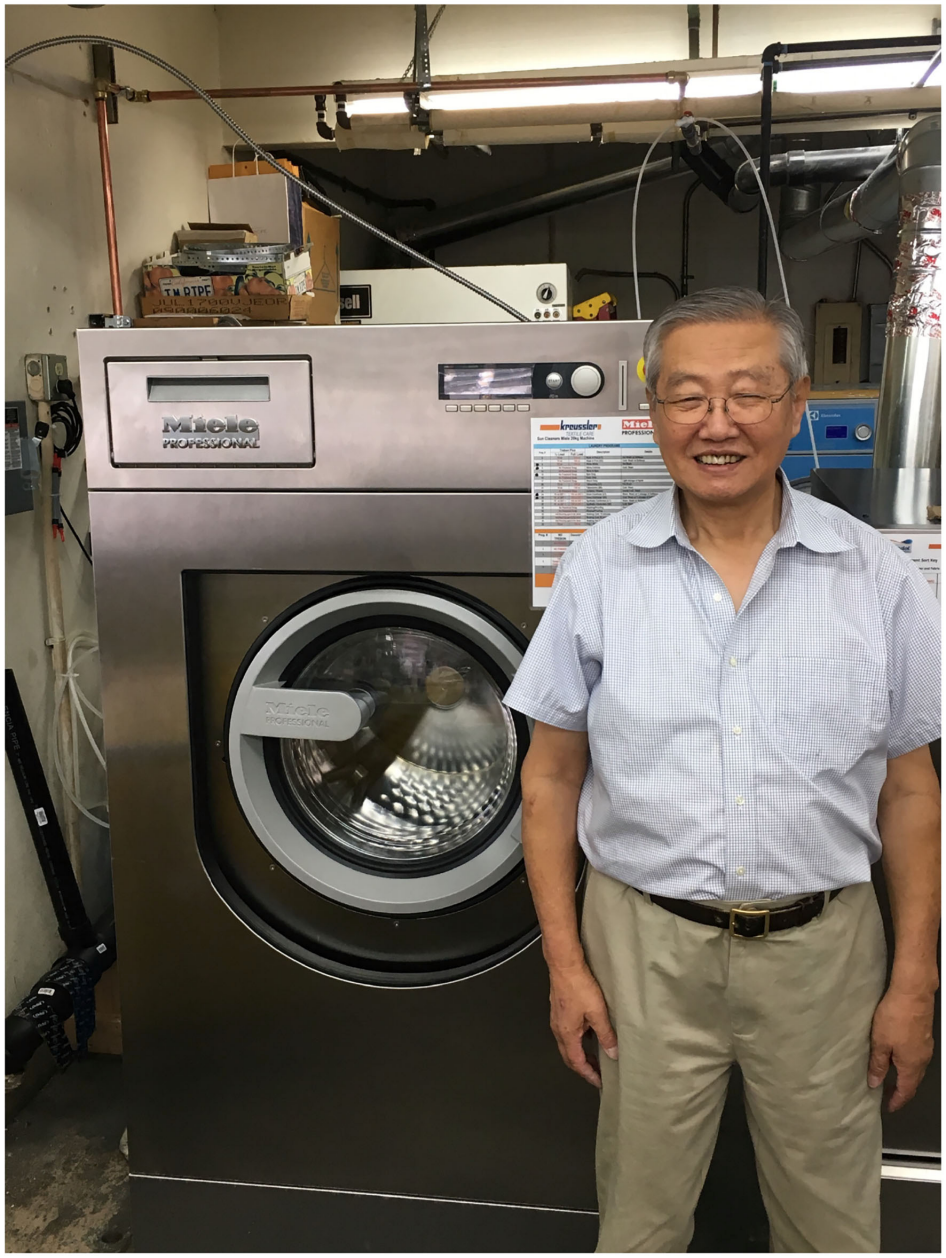
Photo of the owner at one of the dry cleaner shops participating in the KIng County, WA transition program. Credits to Tae Park (Sun Cleaners) and the Hazardous Waste Management Program in King County, Washington, USA.

The program also decided that pursuing a signage regulation would be time-consuming and have little impact. Therefore, the program initiated a pilot project in which dry cleaners would be reimbursed $20,000 if they switched from PERC to PWC (71). The program reserved the option to implement equipment demonstrations, if necessary. Figure 1 depicts a photo of one of the participating dry cleaning shops in the transitioning program.

The strategy to promote the adoption of PWC in King County, WA, USA is described in detail in a technical report (71). Ultimately, the initiative's success hinged on the credible scientific information about PWC already gathered by other programs and the participation of a local Korean-owned vendor, who had become a dealer for Miele PWC equipment. This vendor was established and trusted in the local Korean dry cleaning community because they were already supplying solvents, equipment, and other materials to the industry. The program used an equity and social justice lens in its intervention. Interactions with the dry cleaning community needed to be conducted in a culturally appropriate manner, including working closely with the communities to hear their needs, working with community members (including vendors) to promote the program, soliciting community input on the development of promotional materials, providing materials in their native language, and providing interpretation services, when necessary. Further, the Washington State Department of Ecology also collaborated with the program extensively, providing technical assistance and ensuring that all procedures conformed to local environmental regulatory requirements.

Starting in April 2018, the vendor visited their existing PERC dry cleaning clients to advocate for PWC. The program provided the vendor with promotional materials, which they distributed to the shops. Once the shop owners expressed interest to the vendor, program staff visited the business, usually with a Korean vendor representative. The vendor made introductions to the shop owner and provided interpretation help, as needed. At this visit, program staff administered a survey, inventoried process chemicals, performed leak detection on the PERC machine with a photoionization detector (PID), and provided the paperwork for reimbursement. Of the approximately 65 remaining PERC dry cleaning shops in King County, WA, USA, 27 have taken advantage of the reimbursement program and switched to PWC as of October 2020 (Table 2).

Follow-up surveys were conducted 6 months after each shop transitioned to PWC, and the products used with the new equipment were inventoried. Once shops made the switch, they no longer used a hazardous solvent to clean fabrics and no longer generated organic solvent hazardous wastes. Also, the ancillary process chemicals provided by the PWC vendor (spotting agents, etc.) contain products with ingredients of lower toxicity than those used in PERC operations (74). Although utility data proved difficult to review, two shops that continually flushed cooling water through their PERC machines reduced their water usage and utility bills dramatically after transitioning to PWC. Other studies have documented significantly lower consumption of natural resources (i.e., gas, electricity, and water) when using PWC compared to PERC (46, 76–78). Most shop owners expressed satisfaction with their decision to adopt PWC, with some suggesting that their health had improved (71).

#### Implications for Future Public Health Research and Policy

Dry cleaning businesses have promoted the adoption of alternative technologies in dry cleaning as “green,” in an attempt to change public perception given the increased public awareness of PERC health and environmental issues. Despite marketing efforts to use the technology changes to increase clientele and attract new users, the dry cleaning industry is in a state of decline (92). The industry's financial stress stem from the Great Recession in the US (December 2007–June 2009) and the current recession (2020). This decline also reflects a shrinking customer base because of: (1) changes in the types of fabrics now in common use, many of which do not require dry cleaning; (2) technological advances in residential washing machines and dryers, which allow the cleaning of wool and other delicate fabrics at home; (3) the availability of in-house dry cleaning and “wash & fold services” at several major corporations; and (4) extended telecommuting and other alternative work arrangements in which workers are no longer required to report to an office. Business owners are also retiring, especially immigrants from South Korea. In King County, WA, USA, we are also witnessing considerable consolidation in this industry, insofar as businesses with relatively large facilities and multiple cleaning machines are purchasing neighborhood dry cleaning shops and converting them to drop shops, where fabrics are dropped off by customers and then transported to the central facility for cleaning. Although there are health and environmental benefits to conducting cleaning in a light industrial setting distant from neighborhoods, this development is contributing to the demise of the remaining smaller neighborhood shops that are often owned by financially vulnerable business owners, with significant impacts to their employees, who are disproportionately of Asian and Latin American descent.

Regardless of the economic challenges faced by the dry cleaning industry in the US, dry cleaning shops will continue to operate, especially in larger urban areas, and it is important to ensure the health and safety of the workers and the communities served by these small businesses. Therefore, research efforts should be directed toward understanding and tracking the long-term health effects of exposures to past and present dry cleaning workers, their families, and the surrounding community. Research is especially needed given the lack of health surveillance within these populations of interest. In particular, it would be important to follow the health of workers in dry cleaning shops using alternatives to PERC, where we do not have comprehensive toxicological and human exposure and health information. The impacts of using spot-cleaning agents and other ancillary process chemicals should also be evaluated.

Even if PERC is eliminated in the US dry cleaning industry, it will be necessary to continue to understand the benefits and health impacts from transitioning to alternative solvents and technologies to avoid regrettable substitutions. This feedback loop to assess real-life scenarios of these new technologies, especially as machines age, should be part of the safer alternative strategies, as suggested by OSHA in their guidance Transitioning to Safer Chemicals (Step 7 Evaluate) (93). It is vital to examine the short- and long-term health impacts of PERC exposures on affected workers and communities (3).

A draft risk evaluation report for PERC was released by US EPA in April 2020 and the public comment period closed July 6, 2020 (36). EPA's draft risk evaluation preliminarily found unreasonable risk to workers, occupational non-users, consumers, bystanders, and the environment from certain uses. The primary health risk identified in the draft risk evaluation is neurological effects from short- and long-term exposure. The risk to consumers is from skin exposure to items cleaned with PERC. The agency also found environmental risks to aquatic organisms.

If the final risk evaluation determines that PERC presents an unreasonable risk to human health and the environment, the US EPA would take a risk management action under TSCA. The risk evaluation should be finalized by the end of 2020. Once final and if risks are found, that starts a 1-year clock to propose a risk management rule. The US EPA also has the option to establish regulatory restrictions on the manufacture, processing, distribution, use, or disposal of PERC to eliminate the unreasonable risk. The US EPA is given a range of risk management options under TSCA, including labeling, recordkeeping or notice requirements, actions to reduce human exposure or environmental release, and a ban on the chemical or of certain uses. Like the prioritization and risk evaluation processes, there is an opportunity for public comment on any proposed risk management actions.

## Conclusions

Although local and state policies in the US have played a major role in transitioning dry cleaners from PERC to safer alternatives, identifying safer and more sustainable alternatives to PERC has not been straightforward. Some of these alternatives have been promoted as safe and environmentally friendly, although their effects on human health and the environment may have not been well characterized. Many of the alternative solvents are relatively new products with no established occupational exposure limits (e.g., glycol ethers and Solvon K4). Unfortunately, the search for dry cleaning solvents has resulted in regrettable substitutions, such as the use of *n*-PB. However, with recent improvements in PWC, this technology has become an alternative to PERC that does not use potentially harmful solvents and does not generate organic hazardous waste. To ensure the sustainability of the fabric cleaning industry and the health of workers and nearby communities, continued investment in transition programs and research into safer alternatives to PERC is needed. Lastly, any approach to promoting safer alternatives should account for the unique financial and cultural characteristics of the industry.

## Ethics Statement

Written informed consent was obtained from the individual(s) for the publication of any identifiable images/material in the article.

## Author Contributions

DC and SW conceptualized the idea for the manuscript. DC coordinated collaborators and led the writing and reviews of the manuscript. SW led the writing of the case study, the description of solvent alternatives, and reviews of the manuscript. PJ contributed to the writing of the health section of the manuscript and reviewed the manuscript. KF and AE contributed to the writing of the policy section and case study and reviewed the manuscript. EL contributed to the occupational health and exposure limits sections and the review of the article. All authors contributed to the article and approved the submitted version.

## Conflict of Interest

The authors declare that the research was conducted in the absence of any commercial or financial relationships that could be construed as a potential conflict of interest.
